# Compressive Behaviour of Aluminium Pyramidal Lattice Material-Filled Tubes

**DOI:** 10.3390/ma14143817

**Published:** 2021-07-08

**Authors:** Yingjie Huang, Wenke Zha, Yingying Xue, Zimu Shi

**Affiliations:** 1 School of Physics & Electrical Engineering, Anyang Normal University, Anyang 455000, China; yingjieh@mail.ustc.edu.cn (Y.H.); wkzha@vip.163.com (W.Z.); 2 Key Laboratory of Materials Physics, Institute of Solid State Physics, Chinese Academy of Sciences, Hefei 230031, China; 3 School of Electromechanical and Automotive Engineering, Yantai University, Yantai 264005, China

**Keywords:** multilayer structure, porous materials, aluminium pyramidal lattice material-filled tubes, mechanical properties, energy absorption

## Abstract

This study focuses on the uniaxial compressive behaviour of thin-walled Al alloy tubes filled with pyramidal lattice material. The mechanical properties of an empty tube, Al pyramidal lattice material, and pyramidal lattice material-filled tube were investigated. The results show that the pyramidal lattice material-filled tubes are stronger and provide greater energy absorption on account of the interaction between the pyramidal lattice material and the surrounding tube.

## 1. Introduction

Thin-walled tube structures act to buffer energy and as energy-absorbing components, have a wide range of potential applications in transportation and aerospace engineering [1,2,3]. A thin-walled tube filled with a cellular structure is used to improve the bearing capacity and energy absorption characteristics of the structure. Its lightweight characteristics play an important role in reducing energy consumption and reducing air pollution [4,5,6]. Aluminum foam has the characteristics of low density, high porosity, high specific strength, and high specific stiffness, which is an ideal energy absorbing material [7,8]. In recent years, researchers have attempted to improve the mechanical and energy absorption properties of thin-walled metal tubes and aluminum foam by filling the metal tube with aluminium foam [6]. The results show that the combined structure has a more stable energy absorption process and higher energy absorption efficiency [9,10,11,12,13]. Taherishargh assessed the effect of temperature on the mechanical behaviour of aluminium-alloy foam-filled empty tubes and found that aluminium-alloy foam-filled tubes exhibited improved mechanical properties compared to empty steel tubes at each temperature tested [12]. Niknejad and Rahmani conducted theoretical analysis and an experimental study on hexagonal tubes filled with polyurethane foam and proposed a new theoretical model for the analysis thereof [14]. Duarte et al. studied the material properties of closed cell aluminium foam embedded in a single metal tube [9]. Cao investigated the performance of aluminium foam when used in an aluminium alloy thin-walled structure through uniaxial quasi-static compression tests and numerical simulation [15]. In short, some research results on aluminum foam-filled thin-walled structures have been obtained.

Mechanical properties are important indices guiding the choice of the application of structural materials. Lattice metallic materials are a type of new cellular material emerging in recent years, which have excellent specific stiffness and specific strength and have been applied in transportation structures for lightweight and protective structures due to their excellent energy absorbing ability [15,16,17]. The excellent features make them good filling materials in tandem with structural-grade tubes. However, according to the literature, the most common internal filler material is aluminium foam, and research into the mechanical properties of aluminium lattice material-filled tubes is sparse, despite their potentially excellent performance. The purpose of the present research was to fabricate and investigate the mechanical properties of aluminium pyramidal lattice material-filled tubes. The results show that the aluminium pyramidal lattice material-filled tubes were stronger and provided greater energy absorption than the empty tube and lattice material due to the interaction between the lattice material and the empty tube.

## 2. Experimental Work

### 2.1. Structural Design

**Figure 1:**
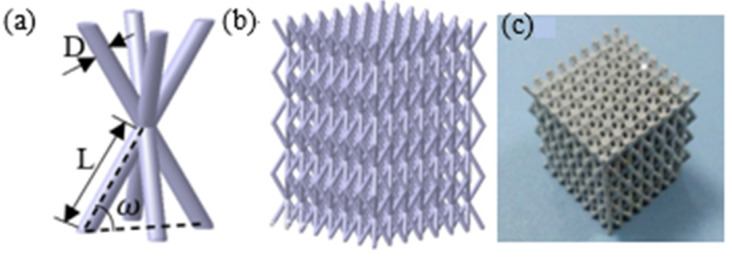
(**a**) The unit cell of the 3-D pyramid lattice material, (**b**) periodic 3-D pyramid lattice material, (**c**) aluminium pyramid lattice material.

The unit cell of the 3-D pyramidal lattice material including eight structural sub-units is shown in Figure 1a: it is characterised by its top-to-top pyramidal structure. The periodic 3-D pyramidal lattice material can be obtained by extension through CATIA V5 R20 software, as shown in Figure 1b. To describe the structural features, three structural parameters are used: strut length *L*, diameter *D*, and inclination angle *ω* between the strut and the horizontal plane (Figure 1a). For pyramidal lattice material, the effect of the strut length or inclination angle is realised by changing the relative density of the lattice samples [18]. While the inclination angle is constant, the mechanical properties are related to the aspect ratio *L*/*D* [19]. To keep the sample size constant, the included inclination angle *ω* and strut length *L* were kept unchanged in the present research, and the relative density was changed by changing the diameter *D*. Several samples were designed (Table 1). The relative density of the pyramidal lattice materials is given by

*ρ* = *ρ*_s_/*ρ*_0_ (1)

where *ρ*_s_ and *ρ*_0_ are the apparent density and the density of aluminium respectively, where *ρ*_s_ is determined by the mass and volume of the pyramidal lattice materials.

### 2.2. Fabrication of Lattice Samples

**Figure 2:**
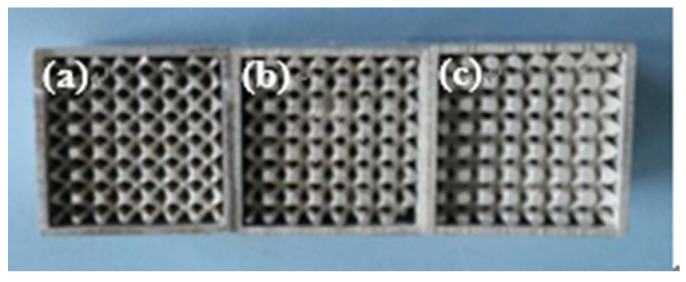
Pyramid lattice material-filled tube with different relative densities of lattice material: (**a**) 0.28; (**b**) 0.33; (**c**) 0.41.

The pyramidal lattice materials used in the present research were made of commercial-grade aluminium, and the fabrication method was similar to that used when producing other lattice structures [20]. First, the pyramidal lattice materials were designed through CATIA software, and the corresponding photosensitive resin patterns were prepared using a 3-D printer. The patterns were put in a container and then plaster was poured onto them. The patterns were removed, and solid plaster moulds were formed with a pyramidal lattice material cavity after drying and baking. Molten aluminium was then poured into the mould, and compressed air was fed to the mould to ensure that the molten aluminium filled all the fine cavities within the mould. Finally, the plaster shell mould was spray-washed, allowing collapse, and the plaster shell removed, leaving an aluminium lattice structure as shown in Figure 1c. The 6063 aluminium alloy empty tube specimens with length, width and height of 20, 20 mm, and 27 mm respectively, and the wall thickness of 1 mm were filled with pyramidal lattice material. The pyramidal lattice material was squeezed into the 6063 aluminium alloy empty tube by a press-fit technique, and the resulting pyramidal lattice material-filled tubes are shown in Figure 2. The principle of thermal expansion and cold contraction was used to obtain an interference fit between pyramidal lattice material and 6063 aluminium alloy tube.

### 2.3. Mechanical Measurement

Mechanical measurements were conducted in a material measuring system (Instron 3369) at a rate of 2 mm/min. To avoid the influence of size effects on the mechanical properties of lattice materials, the number of unit cells in a sample was 7/7/4 in the *X*/*Y*/*Z*-directions, respectively. The experiments were conducted on square samples based on three different geometrical configurations including pyramidal lattice materials, empty tubes, and pyramidal lattice material-filled tubes. Each compression test was conducted along the *Z*-axis of the pyramidal lattice materials. Three samples were tested for each structure, and the arithmetic mean of the measured data was taken as the final value representing each group of samples.

## 3. Results and Discussion

### 3.1. Effects of Structural Parameters on Compression Mechanical Behaviour

**Figure 3:**
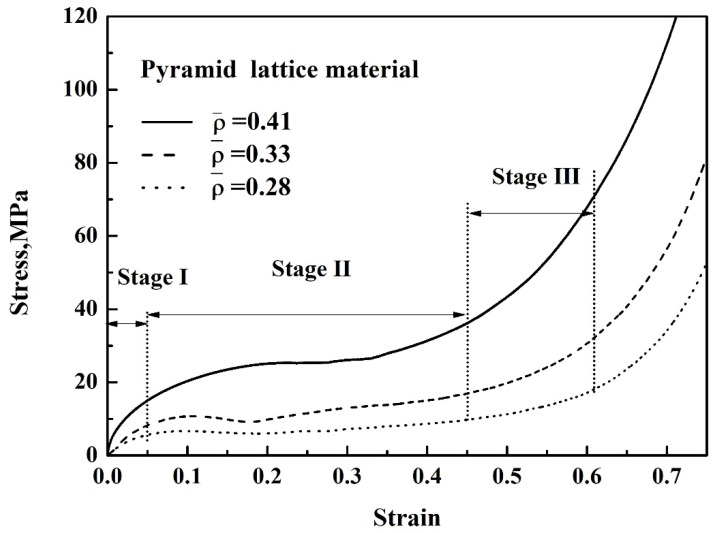
Stress–strain curves of the pyramid lattice material with different relative densities.

**Figure 4:**
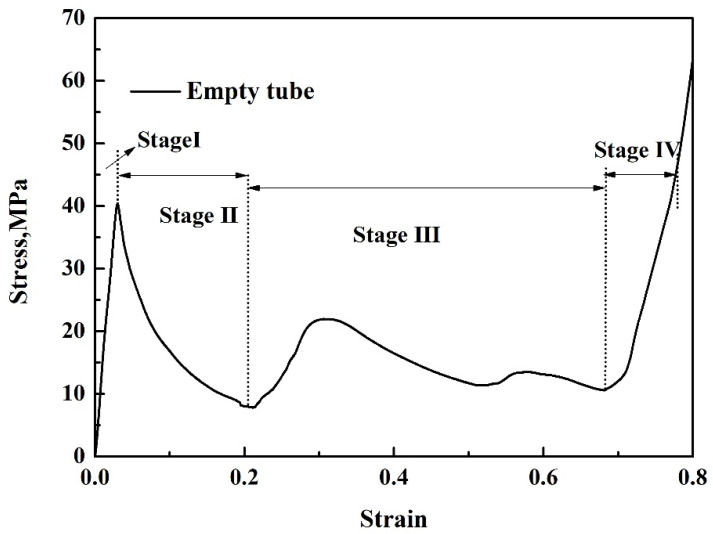
Stress–strain curves of the 6063 aluminium alloy empty tube.

**Figure 5:**
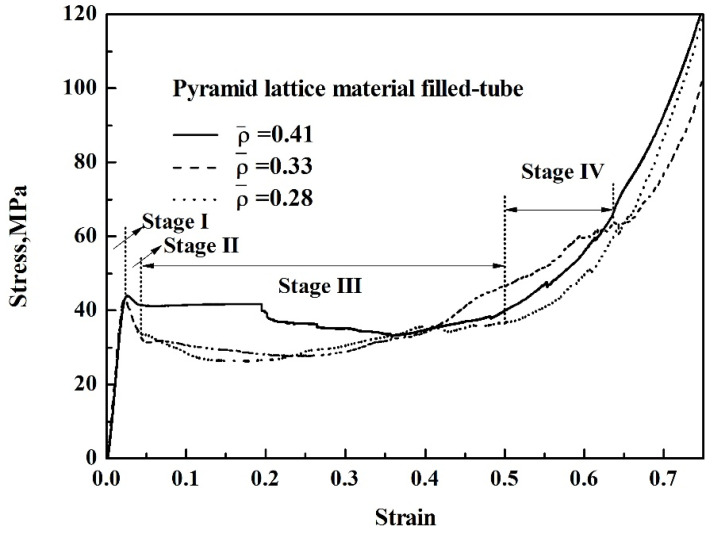
Stress–strain curves of the pyramid lattice material-filled tube with different relative densities.

As shown in Table 1, when the strut length *L* and inclination angle *ω* are constant, the relative density decreases with the increase of the diameter *D*. Figure 3 shows that for typical quasi-static stress–strain curves of Al pyramidal lattice materials, similar to other common porous or cellular metallic materials, the stress–strain behaviour of Al pyramidal lattice material exhibits a three-region characteristic including linear elastic, plateau, and densification regions [21,22]. Unlike the tensile-dominant lattice material, there is no softening beyond the linear elastic region of the stress–strain curve. Although the linear portion of the stress–strain characteristic for such lattice materials is not as large as that of stretch-led lattice materials, it has a stronger linear relationship than that of an open-cell aluminium foam. This may be due to the irregular arrangement of open-cell aluminium foams causing local buckling of some cells at lower stresses. It can be seen from Figure 4 that the whole compression deformation process of an empty tube can be divided into five regions: (A) elastic region, (B), the second region where the highest stress-drop occurs, (C) the third region of stress oscillation, and (D) the region in which the densification of the empty tube occurs. This is caused by buckling of the thin-walled 6063 aluminium alloy empty tube during axial compression. It is worth noting that the stress–strain behaviours of the pyramidal lattice material-filled tubes exhibit dissimilar features to the empty tube and are significantly different from the Al pyramidal lattice materials as shown in Figure 5. The stress–strain behaviour of the Al pyramidal lattice material exhibits a four-region characteristic: a linear elastic region, softening region, a plateau, and a densification region. 6063 aluminium alloy tubes play a leading role in the linear elastic zone, so the stress decreases when the empty tube is rendered unstable. With increasing strain, due to the supporting action of the internal lattice material, the empty tube can no longer buckle, so the stress platform does not appear as a peak within the plateau region. In the elastic region, the stress–strain curves of pyramidal lattice material-filled tubes with three different relative densities are almost coincident. Moreover, the stress–strain curves of pyramidal lattice material-filled tubes with relative densities of 0.33 and 0.28 are similar; however, the plateau region of pyramidal lattice material-filled tubes with a relative density of 0.41 bears a greater stress and is much more stable (especially when the strain is less than 20%), which can be attributed to the interaction between the empty tube and the struts of the Al pyramidal lattice materials. When the relative density of the lattice material is low, the lattice material is not dense enough to prevent deformation of the empty tube.

**Figure 6:**
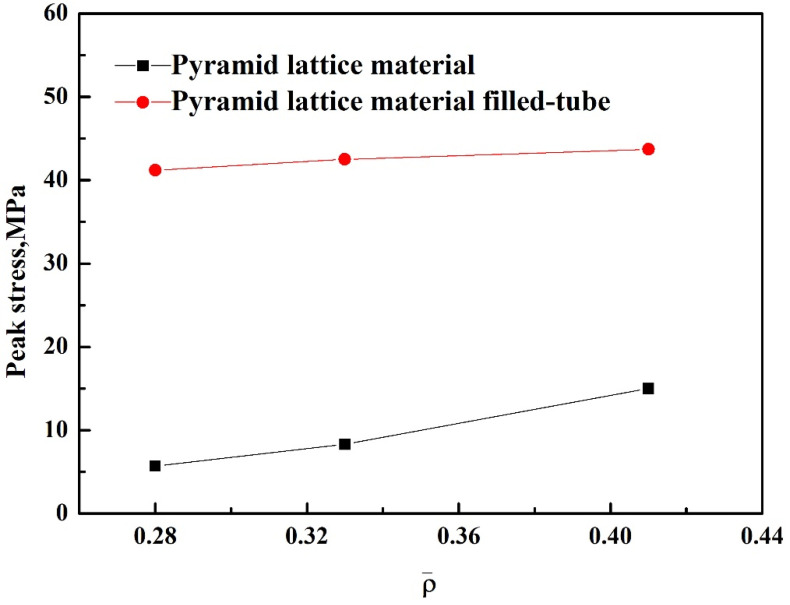
Peak stress of the pyramid lattice material and pyramid lattice material-filled tube with different relative densities.

Due to the lack of a pronounced yield stress in the pyramid lattice materials, the peak compressive strength was determined at an offset strain of 5% [21], and the peak compressive strengths of pyramid lattice material filled-tubes and the empty tube were taken as the initial peak stress. It can be seen from Figure 6 that the peak compressive strength of the lattice material and that when it is filling a tube increases with its relative density; however, the increase in the peak compressive strength of the lattice material-filled tube is small, which is attributed to the fact that both the lattice material and the lattice material-filled tube are in the elastic zone before the peak compressive strength is reached, and the support effect on the lattice material is small.

**Figure 7:**
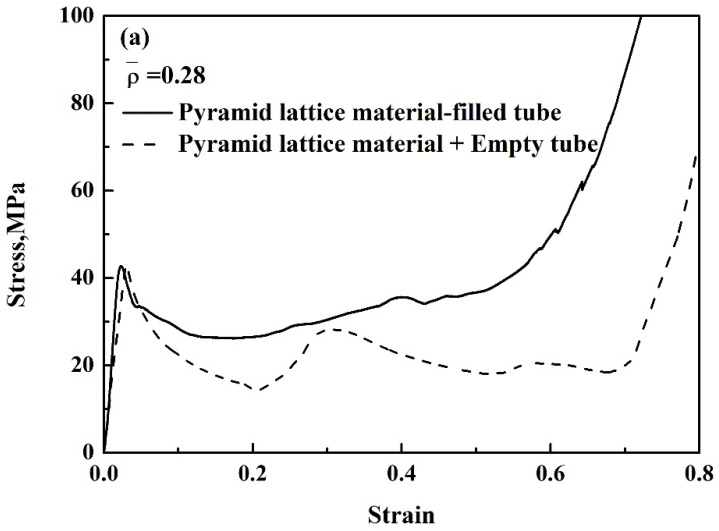

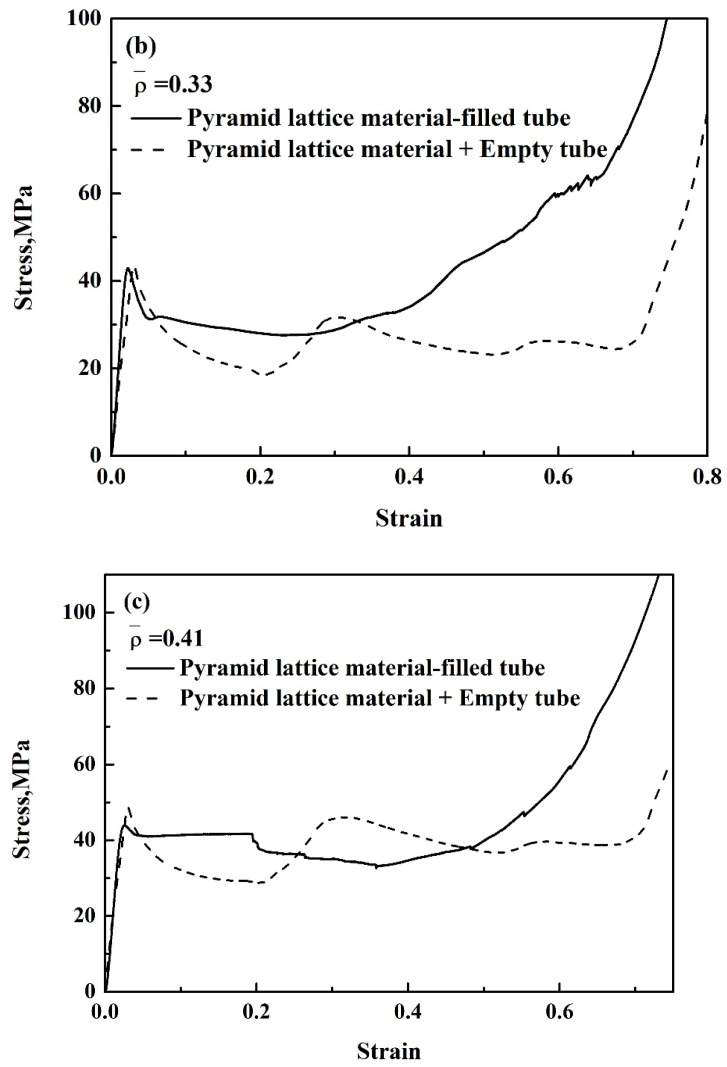
Stress–strain curves of the pyramid lattice material-filled tube and the pyramid lattice material + empty tube with lattice material of different relative densities: (**a**) 0.28, (**b**) 0.33, (**c**) 0.41.

**Figure 8:**
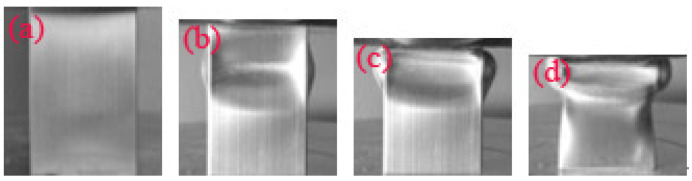
Typical deformation of an empty tube at a strain of (**a**) 2.6%; (**b**) 10%; (**c**) 20%; (**d**) 30%.

**Figure 9:**
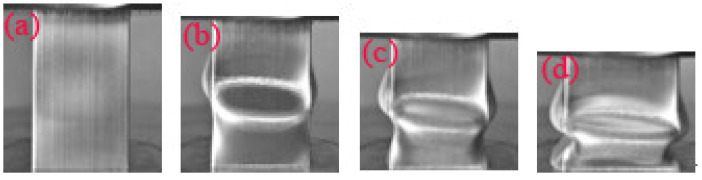
Typical deformation of the pyramid lattice material-filled tube with a relative density of the lattice material of 0.33 at a strain of (**a**) 2.6%; (**b**) 10%; (**c**) 20%; (**d**) 30%.

**Figure 10:**
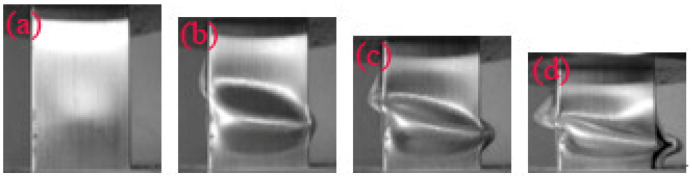
Typical deformation of the pyramid lattice material-filled tube with a relative density of the lattice material of 0.41 at a strain of (**a**) 2.6%; (**b**) 10%; (**c**) 20%; (**d**) 30%.

**Figure 11:**
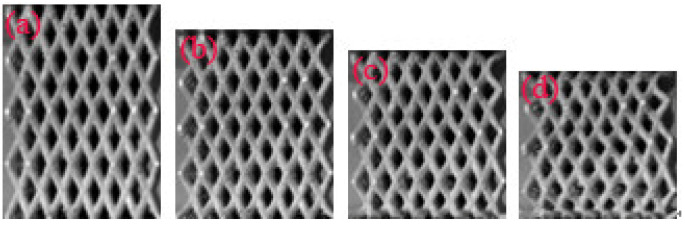
Typical deformation of the pyramid lattice material with a relative density of 0.41 at a strain of (**a**) 2.6%; (**b**) 10%; (**c**) 20%; (**d**) 30%.

Figure 7 shows the engineering stress–strain curves of the pyramidal lattice material-filled tube and the sum of the stresses on the pyramidal lattice material and empty tube. Figure 8, Figure 9 and Figure 10 show that a pyramidal lattice material-filled tube is stiffer than the counterpart empty tube. In the elastic region, the stress–strain curve of the pyramidal lattice material-filled tubes almost coincides with that of the empty tubes. This is because there is no bending deformation in the pyramidal lattice material, and it is the empty tube that mainly bears the load due to its much higher strength; thus, the response of the pyramidal lattice material-filled tubes reflects the empty tube’s behaviour, giving two overlapped stress–strain curves. With increasing strain, the empty tube will buckle and appear concave or convex. The pyramidal lattice material in the interior cannot completely prevent this, therefore, the stress–strain curve trends downwards, but it also plays a certain role, so that the stress–strain curve will plateau. In the plateau region, the stress of pyramidal lattice material-filled tubes is higher than that of the empty tube and Al lattice material, and the stress on the composite material is much greater than the sum of the stresses on the 6063 Al alloy tube and pure Al lattice material (Figure 7). This is due to the constraining effect of the empty tube on the deformation struts and the friction between the pyramidal lattice material and the empty tube; thus the stress and stability of the empty tube are improved. At the same time, it can be seen from the deformation pattern of the pyramidal lattice material that there is an obvious lateral expansion during the compression process (Figure 11), and the incorporation of pyramidal lattice materials within the empty tube significantly enhance its mechanical properties under compression. When the relative density of the pyramidal lattice material is 0.41, it can be seen from Figure 7c that the sum of the stresses on the pyramidal lattice material and empty tube is higher than that on the pyramidal lattice material-filled tube over the range of strains from 0.3 to 0.5. This is because the pyramidal lattice material with a relative density of 0.41 is much stronger than the empty tube and the interaction becomes stronger. The lateral expansion of the lattice material caused the rupture of the empty tube (Figure 10c).

### 3.2. Effects of Structural Parameters on the Average Crushing Force

The average crushing force is given by:

(2) $$F_{m}=1/s\int_{0}^{s}F\left(s\right)ds$$

where *s* is the compression displacement, *F*(*s*) denotes the force at displacement *s*.

**Figure 12:**
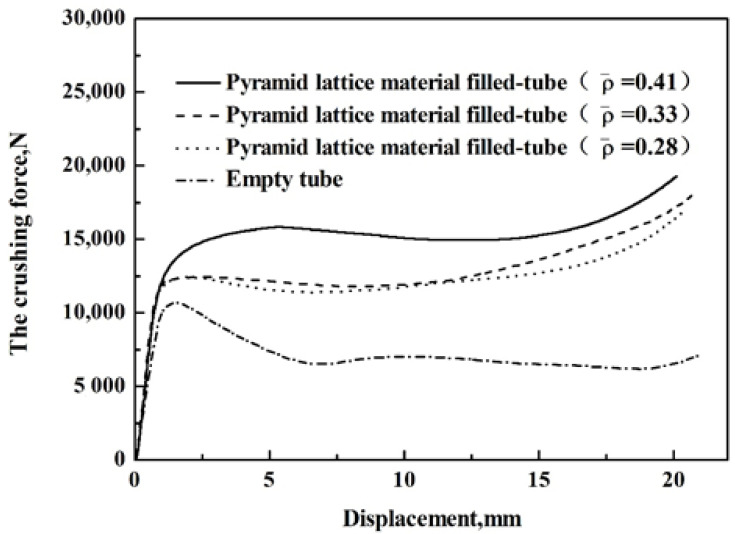
The average crushing force-displacement curves of the pyramid lattice material with different relative densities.

The average crushing force-displacement curves of pyramidal lattice material-filled tube and empty tube are shown in Figure 12. The average crushing force-displacement curves of the pyramidal lattice material-filled tube and empty tube are similar. In the initial stage, the average crushing force increased rapidly with the increase of displacement and soon reached the peak value. After the peak value, there is a long platform area; however, after the average crushing force of empty tube reached the peak value, a significant decline occurred, caused by the rapid decrease in load due to the buckling of the pipe wall. With the increase in the relative density of the lattice material, the average crushing force of the pyramidal lattice material-filled tube increased.

### 3.3. Effects of Structural Parameters on Energy Absorption

Energy absorption per unit volume is important in the design of such cellular materials and can be given by

(3) $$W=\int_{\varepsilon_{1}}^{\varepsilon_{2}}\sigma d\varepsilon$$

where $\varepsilon_{1}$ and $\varepsilon_{2}$ are the compressive strains, and σ is the compressive stress.

Energy absorption per unit mass is important in the design of such cellular materials and can be given by

(4) $$W=\frac{\int_{s_{1}}^{s_{2}}Fds}{m}$$

where $s_{1}$ and $s_{2}$ are the compressive displacement, m is the mass, and F is the compressive force.

**Figure 13:**
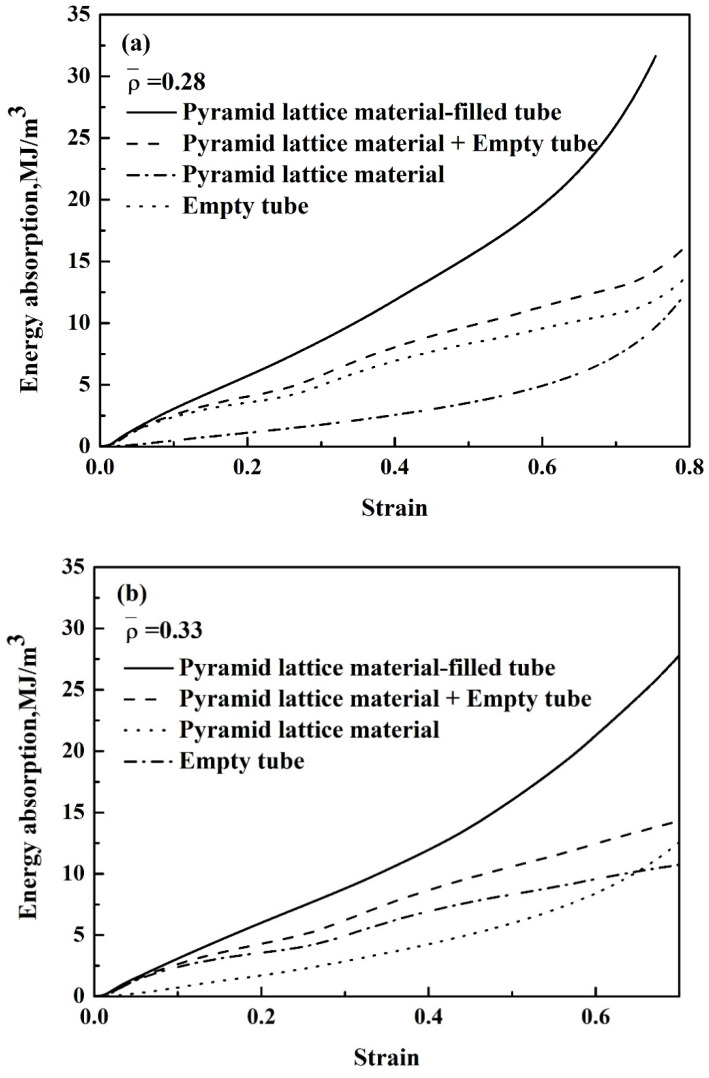

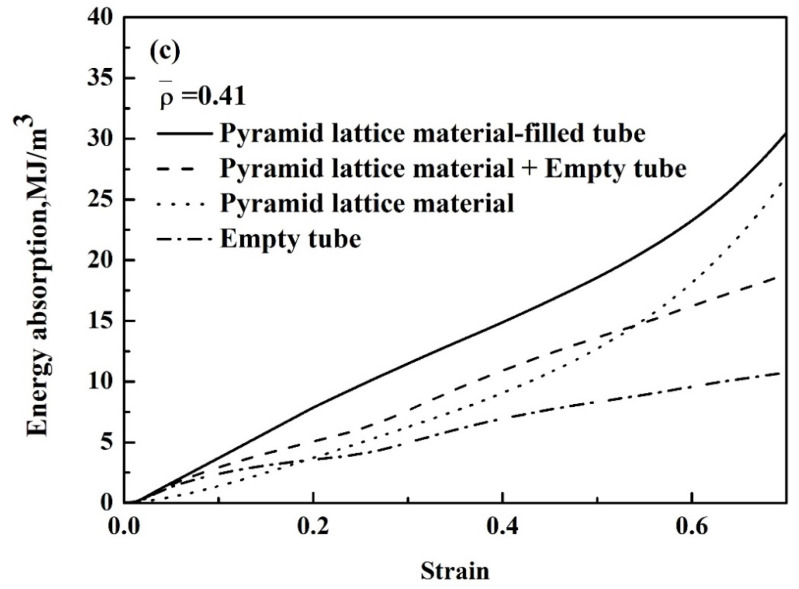
Energy absorption per unit volume of the pyramid lattice material-filled tube with lattice materials of different relative densities (**a**) 0.28, (**b**) 0.33, (**c**) 0.41.

The presented stress–strain behaviour directly affects the energy absorption per unit volume of the pyramidal lattice material, empty tube, and pyramidal lattice material-filled tubes. Up to strains of 5%, for all samples, the energy absorption per unit volume is similar, while when increasing the strain, the difference between samples increased as shown in Figure 13a–c. With increasing strain, the energy absorption per unit volume of pyramidal lattice material-filled tubes increases to above that of the pyramidal lattice material and empty tube and exceeds the sum thereof. For example, at a strain of 0.5, the sum of the energy absorbed by the pyramidal lattice material-filled empty tube is 11.1 MJ/m^3^, but the energy absorbed by the corresponding pyramidal lattice material-filled tube is 15.5 MJ/m^3^ (Figure 13a). The energy absorption per unit mass of the pyramidal lattice material, empty tube, and pyramidal lattice material-filled tubes at a strain of 0.5 is shown in Table 2. From the table, it is clear that the pyramidal lattice material-filled empty tube provided a significantly higher energy absorption per unit mass than the sum of the energy absorption per unit mass in the pyramidal lattice material and empty tube. When the relative density of the lattice material was 0.41, the energy absorption per unit mass of lattice material-filled was 200 J/g, and the sum of the energy absorption per unit mass for the pyramidal lattice material and empty tube was 187 J/g. The pyramidal lattice material inside the tube helps to absorb more energy and bear a higher compressive load than the empty tube. These results indicate the potential to tailor the geometric features of multi-material structures to achieve excellent strength and energy-absorption properties.

## 4. Conclusions

This study focuses on the mechanical properties of aluminium pyramidal lattice material-filled tubes. It is shown that, unlike empty tubes, the aluminium pyramidal lattice material-filled tubes exhibit four-stage compressive mechanical behaviour. The interaction between lattice material and the empty greatly decreases the stress drop and there is no obvious stress fluctuation. When the relative density of the lattice material is 0.28 and the strain is 0.3, the energy absorption of the aluminium pyramidal lattice material-filled tubes increases by 26% compared with the sum of the lattice material and the empty tube. When the relative density of the lattice material is 0.41 and the strain is 0.2, the stress of the aluminium pyramidal lattice material-filled tubes increases by 24% compared with the sum of the lattice material and the empty tube. The results also showed that both the mechanical behaviour and energy absorption of the pyramidal lattice material-filled tubes improve as a result of the interaction between the pyramidal lattice material and the empty tube. These results provide guidelines for engineering and developing new thin-walled tube structures with enhanced mechanical properties for a wide range of applications and further development of multi-material structures.

## Figures and Tables

**Table 1:** Structural parameters of the pyramidal unit cell.

Sample Number	*D* (mm)	*ω* (°)	*L* (mm)	*ρ*
**1**	0.8	60	3	0.28
**2**	0.9	60	3	0.33
**3**	1.0	60	3	0.41

**Table 2:** Energy absorption per unit mass of the pyramid lattice material-filled tube, lattice material, and empty tube.

Sample	The Relative Densities of Lattice Material	Energy Absorption (J)	Energy Absorption per Unit (J/g)
Lattice material	0.28	29	5.8
Lattice material	0.33	52	7.4
Lattice material	0.41	107	11.2
lattice material-filled tube	0.28	167	15.2
lattice material-filled tube	0.33	175	14.5
lattice material-filled tube	0.41	200	13.3
Empty tube		80	13.3

## Data Availability

The data presented in this study are available in insert article.
